# Structure-based virtual screening and *in vitro* validation of inhibitors of cyclic dinucleotide phosphodiesterases ENPP1 and CdnP

**DOI:** 10.1128/spectrum.02012-23

**Published:** 2023-12-14

**Authors:** Akshay Rohilla, Alok Kumar Singh, Benjamin Koleske, Geetha Srikrishna, William R. Bishai

**Affiliations:** 1 Department of Medicine, Johns Hopkins University School of Medicine, Baltimore, Maryland, USA; 2 Center for Tuberculosis Research, Johns Hopkins University School of Medicine, Baltimore, Maryland, USA; Indian Institute of Science Bangalore, Bangalore, Karnataka, India

**Keywords:** *Mycobacterium tuberculosis*, cyclic dinucleotide phosphodiesterase, immune evasion, host-directed therapy

## Abstract

Cyclic GMP-AMP synthase (cGAS) and stimulator of interferon genes (STING) are intracellular mediators of innate immune responses to cytosolic pathogen-derived and host DNA. STING agonists designed to mimic the natural host STING ligand, 2′,3′-cyclic GMP-AMP (cGAMP), are promising immunotherapeutic tools for infectious diseases and solid tumor immunotherapy. We previously characterized CdnP (Rv2837c), a specific phosphodiesterase (PDE) deployed by *Mycobacterium tuberculosis* (*M.tb*), as an enzyme that blunts host immunity by directly cleaving bacterial-derived c-di-AMP and host-derived 2′,3′-cGAMP. We hypothesized that small molecule inhibitors of bacterial and host cyclic dinucleotide PDEs, namely CdnP and the endogenous host PDE, ENPP1, might potentiate the STING pathway and act as host-directed therapies (HDTs) for tuberculosis. To this end, we employed virtual screening of an NCI compound library customized for improved oral drug properties to identify potential inhibitors of CdnP and ENPP1. Compounds identified *in silico* were tested for their inhibitory activity against purified CdnP and ENPP1. Using biochemical and cell-based assays, we identified compounds with low IC_50_ values against both PDEs. We validated increased cGAS-STING signaling in primary human macrophages exposed to 2′,3′-cGAMP in the presence of a lead ENPP1 inhibitor, E-3 (NCI-14465). Our studies provide a framework for novel HDTs that target the cGAS-STING pathway to promote *M.tb* containment and anti-tumor immunity.

**IMPORTANCE:**

In this paper, we describe novel inhibitors of cyclic dinucleotide phosphodiesterase enzymes from *Mycobacterium tuberculosis* (*M.tb*) (CdnP) and mammals (ENPP1). The phosphodiesterase enzymes hydrolyze cyclic dinucleotides, such as 2′,3′-cyclic GMP-AMP and c-di-AMP, which are stimulator of interferon gene (STING) agonists. By blocking the hydrolysis of STING agonists, the cyclic GMP-AMP synthase (cGAS)-STING-IRF3 pathway is potentiated. There is strong evidence in tuberculosis and in cancer biology that potentiation of the cGAS-STING-IRF3 pathway leads to improved *M.tb* clearance and also improved antitumor responses in cancer. In addition to the identification of novel inhibitors and their biochemical characterization, we provide proof-of-concept evidence that our E-3 inhibitor potentiates the cGAS-STING-IRF3 pathway in both macrophage cell lines and also in primary human monocyte-derived macrophages.

## INTRODUCTION

The discovery of cytosolic DNA sensing by cyclic GMP-AMP synthase (cGAS) and subsequent activation of the stimulator of interferon genes (STING) is a major milestone in our understanding of innate host defenses in response to pathogenic DNA molecules (1 – 3). Although the cGAS-STING pathway evolved as a key host defense mechanism for sensing non-self pathogenic DNA molecules, it also plays a crucial role in inflammatory diseases, anti-tumor responses, and autoimmune pathologies (4 – 6). Cytosolic double-stranded DNA from either pathogen-derived or tumor sources binds and activates cytosolic cGAS to generate the host cyclic dinucleotide (CDN) second messenger cyclic GMP-AMP (cGAMP) (7). cGAMP-mediated STING activation of TBK1 and IKK results in the expression of type I interferon (IFN), TNF-α, and IL-6 (8). While the release of STING-dependent cytokines and chemokines is thought to be the primary means of establishing inflammation and antitumor immunity, the STING pathway also activates host autophagy through a mechanism that is independent of TBK1 activation (9). Immune cell-derived type I IFNs drive anti-tumor immunity against many cancers (10), and STING-deficient animals show increased tumorigenesis and intratumoral administration of small molecule STING agonists eliminating tumors *in vivo* (11, 12). Uncovering immunological consequences of cGAS-STING activation during infections and in the tumor microenvironment has enabled new avenues for therapeutic interventions against cancer and infectious pathogens (13 – 15).

However, the strong anti-tumor responses of STING agonists, including cGAMP, may be dampened by the rapid degradation of cGAMP by ectonucleotide pyrophosphatase/phosphodiesterase I (ENPP1). ENPP1 is a type II transmembrane glycoprotein expressed both as a transmembrane protein with an extracellular catalytic domain and as a secreted extracellular soluble protein (16, 17). Recently, ENPP1 has been shown to serve as a phosphodiesterase that degrades STING ligands, thus attenuating the cGAS-STING pathway and compromising the antitumor activity of STING agonists. Indeed, ENPP1 inhibitors were shown to delay tumor growth when used in combination with STING agonists (18, 19). ENPP1 was shown to promote immune evasion and tumor metastasis following hydrolysis of cGAMP, further substantiating its pro-tumoral role (20). Interestingly, loss of ENPP1 function suppressed metastasis, restored immune infiltration in the tumor microenvironment, and synergized with immune checkpoint inhibition in a cGAS-STING-dependent manner (20). Inhibition of ENPP1 could therefore offer a novel immunotherapeutic approach to overcoming checkpoint inhibitor resistance, especially in non-inflamed “cold” tumors.

The role of the cGAS-STING pathway in host immunity against *Mycobacterium tuberculosis* (*M.tb*) was established when Watson and colleagues showed that *M.tb* DNA was a key intracellular substrate for the cGAS-mediated synthesis of cGAMP (21). We reported that *M.tb*-derived c-di-AMP, a critical damage-associated molecule and a potent STING agonist, avidly engages the host STING pathway to modulate host immunity. In a mouse infection model, we found that *M.tb* overexpressing the diadenylate cyclase gene *disA* (Rv3586) (the sole enzyme needed to generate c-di-AMP) is attenuated for infection due to robust STING activation (22). Subsequently, we reported a key *M.tb* virulence mechanism that thwarts STING-mediated immune responses through the release of a novel multi-functional mycobacterial phosphodiesterase (PDE) known as CdnP (Rv2837c) (23). *M.tb*-derived CdnP shows bifunctional activity: it degrades not only bacterial-derived CDNs (c-di-AMP and c-di-GMP) but also host-derived 2′,3′-cGAMP. The loss of the *cdnP* gene leads to higher levels of c-di-AMP and cGAMP in *M.tb*-infected macrophages as well as a significant loss of virulence in mice due to enhanced STING activation (23). CdnP thus serves as an important immune subversion mechanism deployed by *M.tb* to modulate host immune responses for successful survival and replication within host cells (24 – 27). Inhibition of CdnP offers a promising approach for host-directed therapies (HDTs) to modulate STING-dependent host immunity against infectious pathogens. Indeed, PDE inhibitors are currently being evaluated with the hope of shortening the duration of treatment for tuberculosis (TB) and for the prevention and limitation of both pulmonary pathology and bacterial drug resistance (28).

The established role of the cGAS-STING pathway in promoting anti-tumor and anti-viral immunity has promoted the development of synthetic small molecule STING agonists that mimic the endogenous STING ligand cGAMP, several of which have entered clinical development (29 – 32). However, these first-generation STING agonists are likely to be unsuitable for systemic administration due to the risk of excessive inflammation and tissue toxicity, related to the known role of the STING pathway in autoimmunity (32, 33). Inhibitors of pathogen-derived CdnP or host-derived ENPP1, therefore, offer a means to exploit the cGAS-STING pathway toward the management of bacterial infections and solid tumor immunotherapy while minimizing adverse effects of systemic STING activation (32, 34).

In this study, we carried out a structure-based *in silico* screen on a compound library to identify potential small molecule inhibitors of two CDN PDEs: eukaryotic ENPP1 and *M.tb* CdnP. We performed proof-of-concept experiments to demonstrate enhanced cGAS-STING/IFN-I responses in macrophage models using a lead PDE inhibitor identified in our screen. Thus, our findings reveal several small molecule inhibitors that may augment the cGAS-STING/IFN-I pathway and offer approaches toward novel therapies for TB and solid tumor malignancies.

## RESULTS

### Virtual screening of inhibitors against mammalian ENPP1 and *M. tuberculosis* CdnP

**Fig 1 F1:**
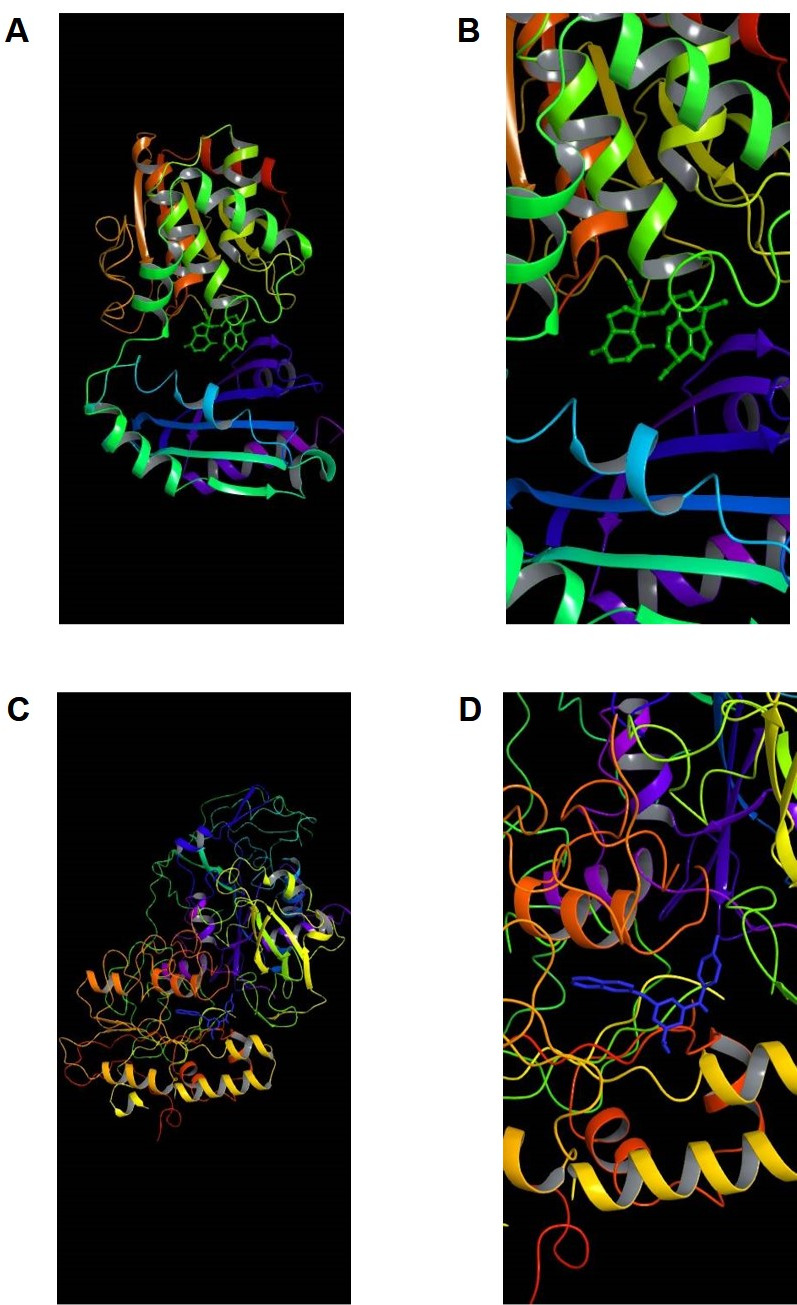
Virtual depiction of CdnP and ENPP1 enzyme ribbon structures with inhibitors docked at the active site. (A) Low and (B) high magnification of inhibitor C-13 (green) docking to the active site of CdnP. (C) Low and (D) high magnification of inhibitor E-3 (blue) docking to the active site of ENPP1.

Identification of inhibitors specific for mouse ENPP1 (protein data bank, PDB ID: 4GTW) and *M.tb* CdnP (PDB ID: 5JJU) was carried out using virtual screening of compounds from a compound library maintained and distributed by the NCI (https://dtp.cancer.gov) consisting of approximately 260,000 small molecules. We initially conducted docking studies on mouse ENPP1, for which the crystal structure was reported in 2012 (17). Subsequently, *in vitro* studies were done using recombinant human ENPP1, which is 80% identical to the murine homolog. 2D simplified molecular-input line-entry system specifications were calculated using OPENBABEL software (https://openbabel.org/docs/dev/Installation/install.html). These files were then submitted to the Free ADME-Tox Filtering Tool (FAF server, https://fafdrugs4.rpbs.univ-paris-diderot.fr/) maintained by the Université Paris Cité for filtering against various drug discovery (35). Using this tool, we sought to optimize the initial compound library for molecules with favorable ADMET (absorption, distribution, metabolism, excretion, and toxicity) profiles and to eliminate duplicate structures and those that violated Lipinski’s rule of five (a set of chemical criteria to predict orally active drugs) (36). This electronic filtration step resulted in a customized library consisting of ~70,000 small molecules. The refined library was then used for *in silico* screening against the active sites of CdnP and ENPP1. Docking studies were carried out using AutoDock 4.2 using parallel grid computing, with grid centers chosen according to the substrate binding pocket as shown in Fig. 1. In addition, we validated our virtual screen by testing known CdnP inhibitors. We tested six CdnP inhibitors reported by Karanja et al. (37) and found that they gave CdnP docking scores ranging from −6.35 to −8.07 kcal/mol (mean docking score −7.11), while ours gave CdnP docking scores ranging from −4.66 to −14.44 kcal/mol (mean docking score −9.45), demonstrating that known CdnP inhibitors docked well in our virtual screen (Fig. S1; Table 1). The 80 strongest hits for binding to each CdnP and ENPP1, as assessed by minimizing the predicted ∆G values (Tables 1 and 2), were selected for further study. Samples of these 160 molecules were obtained from NCI and tested for activity *in vitro*.

**TABLE 1:** IC_50_ values of 16 CdnP inhibitors that showed >30% CdnP inhibition

Inhibitor	NCI number	CdnP docking score in kcal/mol	IC_50_ CdnP in μM (µg/mL)	IC_50_ ENPP1 in μM (µg/mL)	IC_50_ Vero cells in μM (µg/mL)	MW in daltons
C-2	14403	−6.63	90.7 (35)	207 (80)	>259 (>100)	386
C-4	14406	−6.38	125 (50)	125 (50)	>250 (>100)	400
C-5^*a*^	36400	−14.44	28.8 (11.2)	>257 (>100)	154 (60)	389
C-13^*a*^	79195	−4.66	9.66 (4.0)	>241 (>100)	>241 (>100)	414
C-15^*a*^	99864	−11.20	21.5 (8.2)	262 (100)	131 (50)	381
C-19	212394	−5.02	243 (55)	>442 (>100)	>442 (>100)	226
C-25	339934	−11.81	35.3 (17.5)	202 (100)	101 (50)	495
C-28^*a*^	372327	−7.69	15.9 (6.8)	62.0 (26.5)	187 (80)	428
C-29^*a*^ ^*,*^ ^*b*^	378695	−6.69	14.2 (6.0)	19.4 (8.2)	>237 (>100)	422
C-33^*a*^	408099	−12.40	18.8 (7.3)	60.5 (23.5)	>257 (>100)	389
C-34^*a*^	408100	−12.30	13.1 (5.3)	63.3 (25.6)	>247 (>100)	404
C-47	14747	−9.62	239 (85)	>281 (>100)	84.3 (30)	356
C-48^*a*^	31701	−12.72	42.6 (14.6)	292 (100)	29.2 (10)	343
C-65	136090	−8.91	52.8 (25)	73.9 (35)	> 211 (>100)	473
C-67^*a*^	169717	−12.79	41.9 (15)	>279 (>100)	>279 (>100)	358
C-76^*a*^	401266	−7.88	42.9 (10)	>429 (>100)	>429 (>100)	233
Kar-C82^*c*^	–	−6.64	17.5	–	–	–
Kar-C85	–	−8.07	22.0	–	–	–
Kar-C14	–	−6.35	24.8	–	–	–
Kar-C40	–	−7.10	26.4	–	–	–
Kar-C16	–	−7.13	42.6	–	–	–
Kar-C86	–	−7.39	>60	–	–	–

^*a*^ Compounds with CdnP IC_50_ values of 15 µg/mL or less.

^*b*^ Compounds with ENPP1 IC_50_ values of 15 µg/mL or less.

^*c*^ Kar-C82 indicates compound C82 and so on previously reported by Karanja et al. (37).

**TABLE 2:** IC_50_ values of ENPP1 inhibitors that showed >30% ENPP1 inhibition

Inhibitor	NCI	IC_50_ ENPP1 in μM (µg/mL)	IC_50_ Vero cells in μM (µg/mL)	MW in daltons
E-3^*a*^	14465	26.4 (10)	>264 (>100)	379
E-11	82907	245 (100)	>245 (>100)	408
E-12^*a*^	87015	41.1 (12.5)	>329 (>100)	304
E-13	89127	76 (25)	182 (60)	329
E-14	95537	172 (75)	115 (50)	436
E-15	99790	169 (60)	> 282 (>100)	354
E-17^*a*^	107121	15.6 (10)	156 (100)	642
E-23	173694	50.1 (23)	218 (100)	459
E-25^a^	210818	46.6 (12.5)	186 (50)	268
E-27^*a*^	332061	16.3 (5.0)	>326 (>100)	307
E-37^*a*^	667746	44.6 (10)	>446 (>100)	224
E-54^*a*^	76479	13.6 (4.0)	204 (60)	293
E-60^*a*^	99862	9.8 (4.0)	>245 (>100)	410
E-80	637507	186 (75)	>248 (>100)	403

^*a*^ Compounds with ENPP1 IC_50_ values of 15 µg/mL or less.

### Evaluation of CdnP and ENPP1 inhibitors using *in vitro* enzyme assays

#### CdnP inhibitors

**Fig 2:**
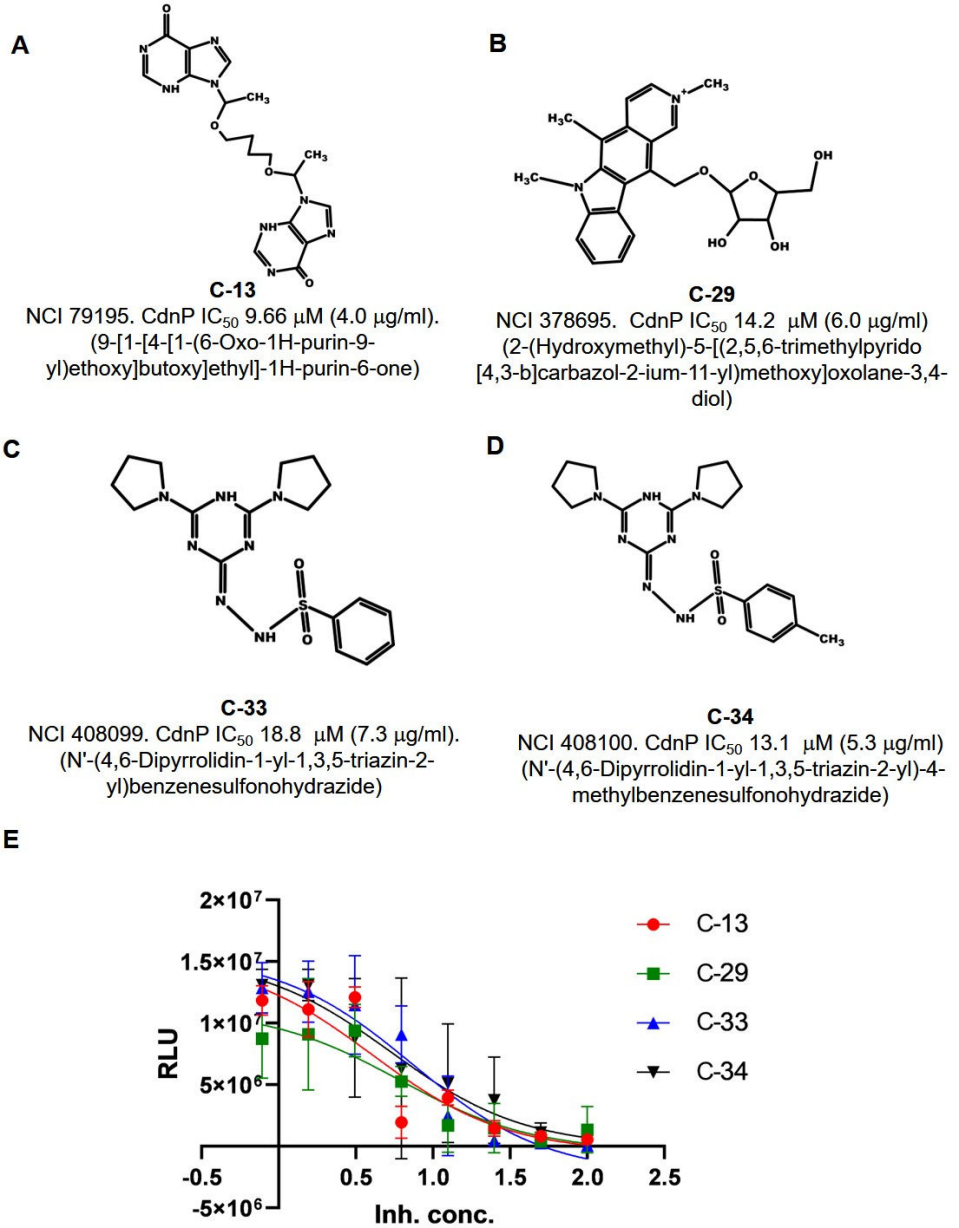
Structures of selected *M.tb* CdnP inhibitors identified in the *in silico* virtual screen. (A–D) Structures, NCI reference numbers, and CdnP IC_50_ values of four selected lead compounds that inhibit *M.tb* CdnP. Inhibitor C-29 showed dual activity against both CdnP and ENPP1. (E) Compounds were tested for their inhibitory potential against purified CdnP protein. AMP, the end-product of the PDE reaction against c-di-AMP, was detected by measuring relative light units (RLU) using our luminescence-based, AMP detection assay.

To evaluate the inhibition of *M.tb* CdnP enzyme activity *in vitro*, we expressed *M.tb* CdnP as a His-tagged protein in *Escherichia coli* and purified as described earlier (23). We standardized the enzyme activity of *M.tb* CdnP by incubating increasing concentrations of c-di-AMP (0.5 to 5 µM) with CdnP and resolving the products AMP and pApA on a high-performance liquid chromatography (HPLC) C-18 column. The products were quantified by measuring the area under the curve (AUC) and correlating these AUCs against an AMP standard of known concentration. This testing with c-di-AMP yielded a *K*_*m*_ of 51.4 µM and a *V*_max_ of 0.5664 µM/min for CdnP. However, since HPLC analysis is not suited for high-throughput analysis, we employed a luminescence-based assay for screening inhibitor compounds. We adapted a commercially available, luminescence-based AMP detection kit (AMP-Glo, see Materials and Methods) assay into a high-throughput format in which purified CdnP was co-incubated with c-di-AMP as a substrate, and levels of the resulting AMP product were measured quantitatively through subsequent conversion to ATP, which enables the added luciferase to generate luminescence. After establishing the assay parameters, the compounds were screened at a fixed concentration of 100 µg/mL. At this concentration, we found that 16 of the 80 CdnP-directed compounds showed more than 30% enzymatic inhibition (Table 1). These 16 compounds were further evaluated for IC_50_ studies at concentrations ranging from ~2.5 to ~300 µM (0.78–100 μg/mL) (Table 1). Of the 16 compounds, we identified five with IC_50_ values of ~19.7 µM (7.5 µg/mL) or less for CdnP: C-13 (IC_50_ 9.66 µM, 4 µg/mL), C-28 (IC_50_ 15.9 µM, 6.8 µg/mL), C-29 (IC_50_ 14.2 µM, 6 µg/mL), C-33 (IC_50_ 18.8 µM, 7.3 µg/mL), and C-34 (IC_50_ 13.1 µM, 5.3 µg/mL). The structures and inhibitory activities of four of these are shown in Fig. 2. Five additional inhibitors (C-5, IC_50_ 28.8 µM, 11.2 µg/mL; C-15, IC_50_ 21.5 µM, 8.2 µg/mL; C-48, IC_50_ 42.6 µM, 14.6 µg/mL; C-67, IC_50_ 41.9 µM, 15 µg/mL; and C-76, IC_50_ 42.9 µM, 10 µg/mL) had IC_50_ values ranging from ~21.0 to 43.0 µM (8–15 μg/mL), and the structures of four of these are shown in Fig. S2.

To evaluate the selectivity of these compounds, we also measured their IC_50_ values for the inhibition of purified human ENPP1. Among the 10 compounds that exhibited an IC_50_ against CdnP of 43.0 µM (15 µg/mL) or less, nine showed IC_50_ values above 53.7 µM (20 µg/mL) for ENPP1, indicating that they were specific for *M.tb* CdnP (Table 1). Compound C-29 had a CdnP IC_50_ of 14.2 µM (6 µg/mL) and an ENPP1 IC_50_ of 19.4 µM (8.2 µg/mL), indicating that it had dual activity for both phosphodiesterases.

#### ENPP1 inhibitors

**Fig 3:**
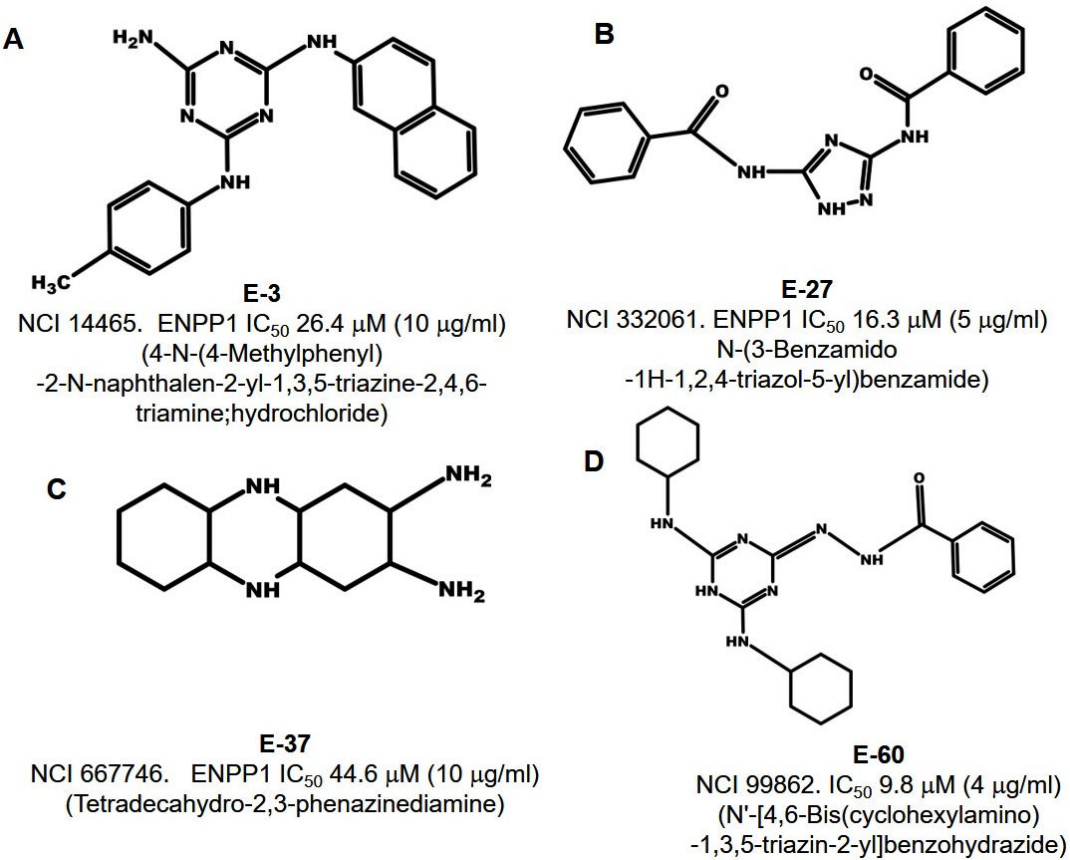
Structures of selected inhibitors of host ENPP-1 identified in the *in silico* virtual screen. (A–D) Structures, NCI reference numbers, and ENPP1 IC_50_ values of four selected lead compounds that inhibit mammalian ENPP1.

Analogous to the above work with CdnP, we first established ENPP1 kinetic parameters using HPLC with 2′,3′-cGAMP as substrate. From our AUC data, we determined a *K*_*m*_ of 63.4 µM and a *V*_max_ of 0.626 µM/min. We then tested 40 of our top *in silico* hits for ENPP1 inhibitors to determine their IC_50_ against purified ENPP1. Using 2′,3′-cGAMP as the substrate in our high throughput luminescence-based assay, we identified 14 of the 40 compounds that showed more than 30% enzymatic inhibition of ENPP1. Among these compounds, 8 of 14 showed ENPP1 IC_50_ values of less than ~47.0 µM (15 µg/mL). These were E-3 (IC_50_ 26.4 µM, 10 µg/mL), E-12 (IC_50_ 41.1 µM, 12.5 µg/mL), E-17 (IC_50_ 15 µM, 10 µg/mL), E-25 (IC_50_ 46.6 µM, 12.5 µg/mL), E-27 (IC_50_ 16.3 µM, 5 µg/mL), E-37 (IC_50_ 44.6 µM, 10 µg/mL), E-54 (IC_50_ 13.6 µM, 4 µg/mL), and E-60 (IC_50_ 4 µg/mL) as shown in Table 2. Additionally, compound C-29 shows dual inhibition of CdnP (IC_50_ 14.2 µM, 6 µg/mL) and ENPP1 (IC_50_ 19.4 µM, 8.2 µg/mL) with IC_50_ values less than ~43–47 µM (15 µg/mL) for both enzymes, as described above and shown in Table 1. The structures of select ENPP1 inhibitors are shown in Fig. 3; Fig. S2.

### Predicted molecular interactions of the lead inhibitor compounds with CdnP and ENPP1

**Fig 4 F4:**
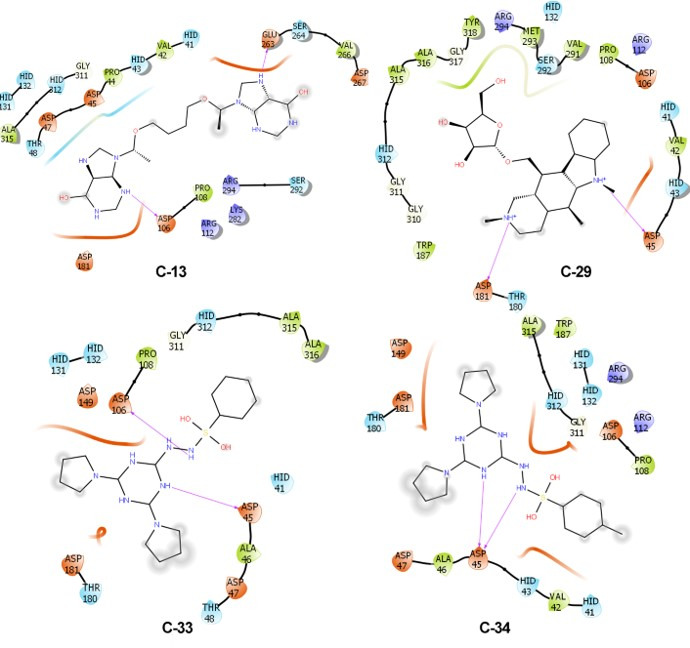
Predicted molecular interactions of four lead inhibitors with *M.tb* CdnP. Molecular docking of inhibitors C-13 (IC_50_ 9.66 µM or 4 µg/mL), C-29 (IC_50_ 14.2 µM or 6 µg/mL), C-33 (IC_50_ 18.8 µM or 7.3 µg/mL), and C-34 (IC_50_ 13.1 µM or 5.3 µg/mL) with the active site of CdnP. Predicted hydrogen bonds are shown by purple arrows, and the hydrophobic surfaces of the inhibitors are shaded in gray. For the enzyme active site, acidic residues and surfaces are colored orange, basic residues are purple, hydrophobic residues are green, and hydrophilic residues and surfaces are blue.

Having validated that many of our hit compounds have low IC_50_ values for CdnP and ENPP1, we conducted more detailed molecular docking studies *in silico* to define specific active site interactions. The high-affinity CdnP inhibitors C-13 (CdnP IC_50_ 9.66 µM, 4 µg/mL), C-29 (IC_50_ 14.2 µM, 6 µg/mL), C-33 (IC_50_ 18.8 µM, 7.3 µg/mL), and C-34 (IC_50_ 13.1 µM, 5.3 µg/mL) oriented well inside the binding pocket of CdnP (Fig. 1A and B, Fig. 4 [Fig F1 F4]). The predicted interactions included hydrogen bonds and salt bridges with key residues in the active site of CdnP. Specifically, one of the purine moieties of C-13 (NCI 79195) is predicted to form a hydrogen bond with Glu263, and the other purine moiety is predicted to form a hydrogen bond with Asp106. In C-29 (NCI 378695), two protonated tertiary amines are predicted to donate hydrogen bonds to Asp45 and Asp181. C-33 (NCI 408099) has a triazine nitrogen that is predicted to hydrogen bond with Asp45 and a benzenesulfonyl hydrazide nitrogen predicted to donate to Asp106. Interestingly, C-34 (NCI 408100), which is closely related to C-33 (differing only by the addition of a para-methyl group off the benzenesulfonyl hydrazide moiety), is predicted to orient differently in the CdnP active site such that Asp106 does not access the hydrazide nitrogens and instead one triazine nitrogen and one benzenesulfonyl hydrazide nitrogen are both predicted to hydrogen bond with Asp45. The work by He et al. to characterize the active site of CdnP showed that enzymatic activity was completely or substantially reduced with the mutation of Asp45, Asp106, or Asp181 (38). Hence, our computational studies depict probable interactions of our lead CdnP inhibitors with vital active site residues, thereby corroborating their inhibitory potential.

**Fig 5:**
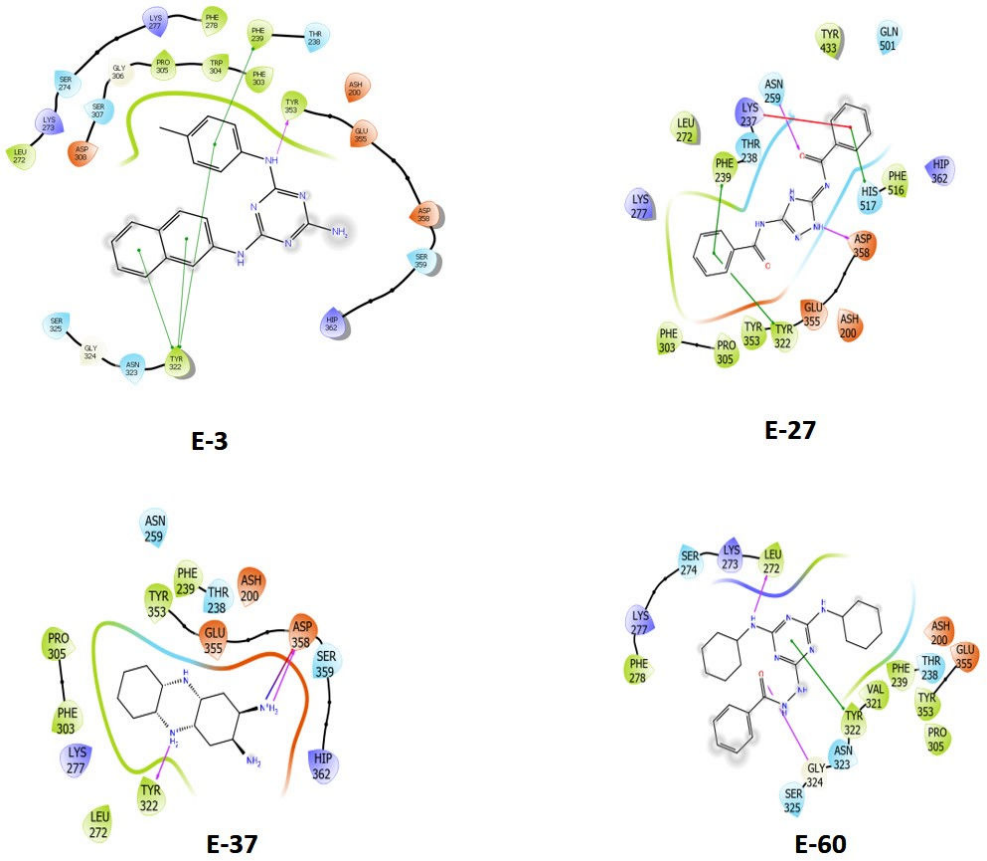
Predicted molecular interactions of the four lead inhibitors with human ENPP1. Molecular docking of inhibitors E-3 (IC_50_ 26.4 µM or 10 µg/mL), E-27 (IC_50_ 16.3 µM or 5 µg/mL), E-37 (IC_50_ 44.6 µM or 10 µg/mL), and E-60 (IC_50_ 9.8 µM or 4 µg/mL) with the active site of ENPP1. Predicted hydrogen bonds are shown with purple arrows, while predicted salt bridges are shown with black arrows. Predicted pi-stacking interactions are shown by green lines, with hydrophobic surfaces of the inhibitors being shaded in gray. Predicted pi-cation interactions are shown with red lines. For the enzyme active site, acidic residues and surfaces are colored orange, basic residues are purple, hydrophobic residues are green, and hydrophilic residues and surfaces are blue.

We also carried out analogous molecular docking predictions of our top ENPP1 inhibitors with the active site binding pocket of that enzyme (Fig. 5). Jansen et al. identified Tyr322, Asp358, and His517 as key nucleotide-binding residues in the ENPP1 active site and additionally found that Asn259 is conserved in phosphodiesterases but absent in pyrophosphatases (16). We focused on four high-affinity ENPP1 inhibitors, E-3 (ENPP1 IC_50_ 26.4 µM, 10 µg/mL), E-27 (IC_50_ 16.3 µM, 5 µg/mL), E-37 (IC_50_ 44.6 µM, 10 µg/mL), and E-60 (IC_50_ 9.8 µM, 4 µg/mL) and found that they are predicted to interact with select catalytic site residues identified by Jansen et al. as well as additional nearby amino acids. The 2-naphthylamine moiety of E-3 (NCI 14465) is predicted to form pi-stacking interactions with Tyr322 and Phe329, while its p-toluidine moiety may form another pi-stacking interaction with Phe329. For E-27 (NCI 332061), a hydrogen bond is predicted between a triazole nitrogen donor and Asp358 and between one benzamide oxygen moiety and Asn259. This benzamide group may also form a pi-pi interaction with His517 as well as a pi-cation interaction with Lys237, while the other benzamide shows predicted pi stacking with Phe239 and Tyr322. For E-37 (NCI 667746), one of the primary amines is predicted to form a hydrogen bond and/or a salt bridge with Asp358, while one of its central secondary amines may hydrogen bond with Tyr322. For E-60 (NCI 99862), one of its cyclohexylamine moieties is predicted to donate a hydrogen bond to the Leu272 backbone, while the benzohydrazide carbonyl oxygen is predicted to accept a hydrogen bond from the Gly324 backbone. Additionally, the central triazine ring is predicted to form pi-stacking interaction with Tyr322 of the binding pocket. Thus, our *in silico* analysis suggests extensive interactions of the lead compounds with key residues that compose the ENPP1 catalytic site.

### Vero cell and CYP450 toxicity studies for CdnP and ENPP1 inhibitor compounds

To assess the potential mammalian cell toxicity of our lead CdnP and ENPP1 inhibitors, we conducted a resazurin-based microtiter plate cell viability assay with Vero (African green monkey kidney epithelial) cells. Increasing concentrations of the inhibitors up to 100 µg/mL were added to 1 × 10^6^ cells per well and incubated for 2 days, at which time resazurin was added. Cell viability was measured by colorimetry on day 3 following inhibitor administration. Of the five compounds that exhibited IC_50_ values of <7.5 µg/mL against CdnP, four inhibitors (C-13, C-29, C-33, and C-34) displayed minimal cytotoxicity against Vero cells at high concentrations (Vero cell growth inhibition IC_50_ >~262 µM, >100 µg/mL, Table 1). Among the eight inhibitors against ENPP1, which showed IC_50_ values of less than ~47.0 µM (15 µg/mL) against ENPP1, five compounds (E-3, E-12, E-27, E-37, and E-60) also showed no toxicity (Vero cell growth inhibition IC_50_ >~262 µM, >100 µg/mL, Table 2). Of note, the dual CdnP/ENPP1 inhibitor, compound C-29, showed no Vero cell toxicity by these metrics. Thus, the majority of our highest activity inhibitors showed Vero cell growth inhibition IC_50_ values >~ 262 µM (>100 µg/mL), underscoring the promise of these compounds for further development.

Since the cytochrome P450 (CYP450) enzyme superfamily acts to detoxify or metabolize countless drugs, we sought to investigate any potential modulation of these enzymes by our inhibitors that could lead to drug–drug interactions. To achieve this, we adapted our high-throughput luminescence-based screening assay to test the effects of the compounds on the activities of several CYP450 isoforms. However, due to the instability of the mitochondrial extracts, we could only detect reproducible activity with one isoform, CYP2C19. We tested the inhibitory activity of the 30 compounds shown in Tables 1 and 2 against CYP2C19 activity, and none of the compounds exhibited inhibition at 100 µg/mL (data not shown).

### An ENPP1 inhibitor compound enhances STING/IRF3/IFN-I signaling in mouse and human macrophages

**Fig 6:**
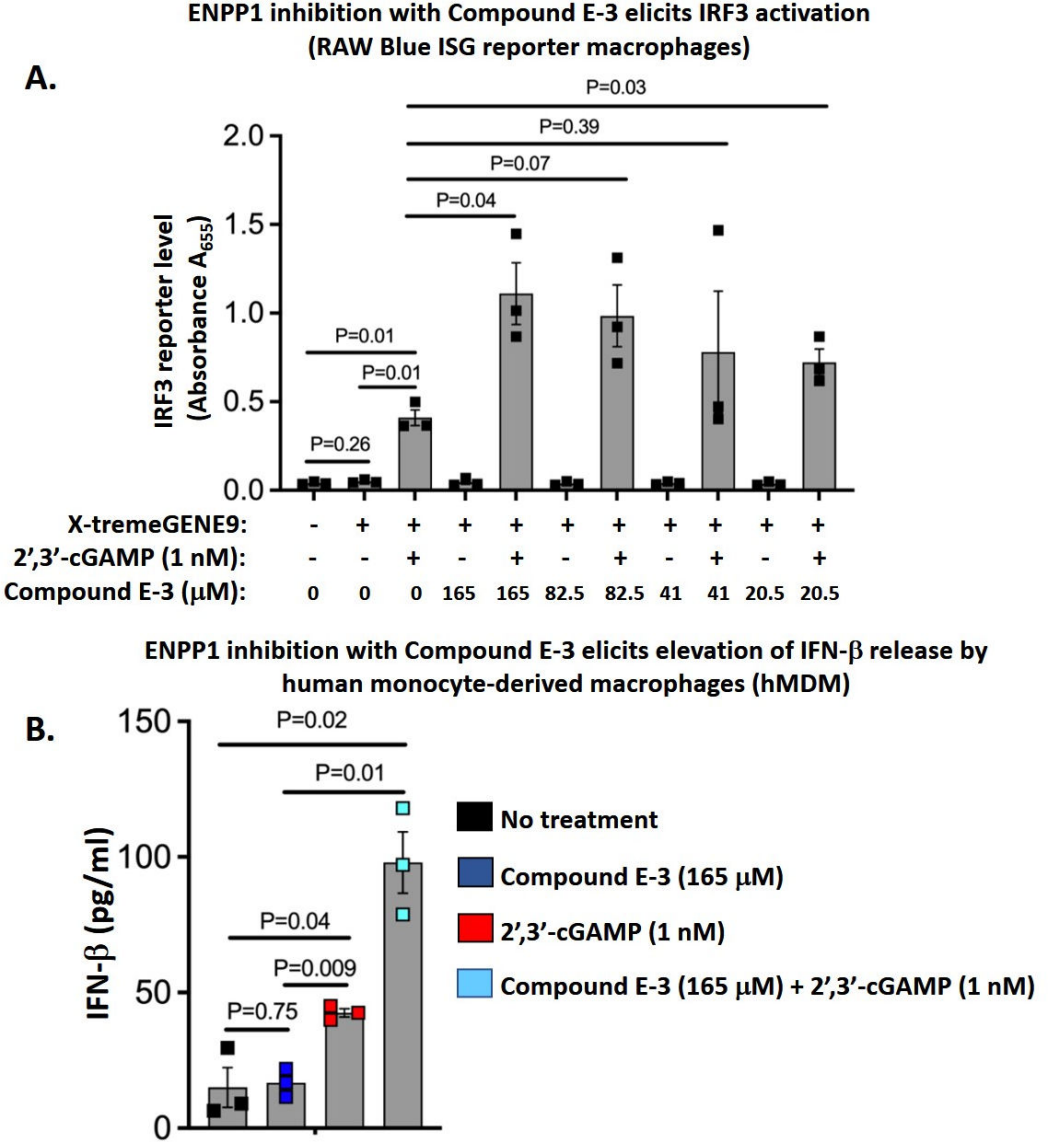
Lead ENPP1 inhibitor, compound E-3, elicits enhanced cGAS-STING-IRF3 pathway activation and type I interferon release in macrophages. (A) Compound E-3 (NCI 14465) elicits enhanced IRF3 activation in RAW-Blue IRF3-SEAP reporter mouse macrophages at 24 h following 2′,3′-cGAMP transfection. Briefly, reporter macrophages were pre-treated with compound E-3 at concentrations varying from 0 to 165 mM (165 mM is 6.2× the ENPP1 IC_50_ and 20.5 mM is 0.75× the IC_50_) and were subsequently transfected with 1 nM 2′,3′-cGAMP using the X-tremeGENE9 transfection reagent. Culture supernatants were collected 24 h after transfection, and SEAP activity was measured by colorimetry in the presence of the QUANTI-Blue detection reagent. (B) Compound E-3 elicits elevated interferon-β (IFN-β) responses in human monocyte-derived macrophages (hMDMs) stimulated with 2′,3′-cGAMP. hMDMs were pre-treated with compound E-3 at 165 µM and subsequently transfected with 2′,3′-cGAMP (1 nM) using the X-tremeGENE9 transfection reagent. At 24 h post-transfection, culture supernatants were evaluated for IFN- β levels via ELISA. All data are presented as mean values ± S.E.M. (*n* = 3 independent biological replicate experiments). Statistical analyses were done using a two-tailed Student’s *t*-test. *P*-values are shown for relevant comparisons.

2′,3′-cGAMP is the natural host ligand for STING, and hydrolysis of 2′,3′-cGAMP by ENPP1 dampens type I IFN release by the cGAS-STING pathway in myeloid cells such as macrophages and dendritic cells. As a proof of concept, we next examined one of our lead ENPP1 inhibitor compounds (E-3, NCI 14465, ENPP1 IC_50_ 26.4 µM, 10 µg/mL, Vero cell growth IC_50_ >~262 µM, >100 µg/mL) for the ability to stimulate the type I IFN pathway in RAW-Blue IRF3 ISG reporter macrophages. These macrophages express secreted embryonic alkaline phosphatase (SEAP) upon IRF3-phospho-activation by STING. Transfection of these macrophages with exogenous 2′,3′-cGAMP (1 nM), produced a fourfold increased IRF induction (0.1 A_655_ units to 0.4 A_655_ units, Fig. 6A; Fig. S3). Next, we performed the assay in the presence of 165 µM (6.2-fold above the ENPP1 IC_50_) to 20.5 µM (0.75-fold above the ENPP1 IC_50_) of compound E-3 (NCI 11465). We observed a 2.75-fold increased signal with 165 µM of E-3 (0.4 A_655_ units with cGAMP alone to 1.1 A_655_ units with cGAMP plus E-3 at 165 µM), which declined in a dose-dependent manner with E-3 concentration (Fig. 6A). Thus, this reporter cell assay demonstrated significantly increased levels of STING-dependent IRF3 activation upon exposure to our E-3 ENPP1 inhibitor.

To validate the observations from the RAW-Blue IRF3 ISG reporter macrophage cell line, we repeated the experiment using primary human monocyte-derived macrophages (hMDMs) transfected with 2′,3′-cGAMP at 1 nM. For the hMDM experiments, we used ELISA to quantify IFN-β release, a known outcome of macrophage STING activation. Untreated hMDMs and hMDMs exposed to 165 µM of compound E-3 (NCI 14465) showed a baseline level of IFN-β release of 10 pg/mL. Stimulation with 1 nM 2′,3′-cGAMP led to a significant 4.5-fold increase in IFN-β release to 45 pg/mL, while stimulation with 1 nM 2′,3′-cGAMP in the presence of 165 µM of compound E-3 (NCI 11465) yielded a further 2.1-fold, statistically significant increase in IFN-β release to 95 pg/mL (Fig. 6B). Thus, in both macrophage cell lines and in primary hMDMs, we validated that compound E-3 (NCI 14465) boosted cGAS-STING signaling in response to an established natural STING agonist, 2′,3′-cGAMP, consistent with the compound’s predicted function as an ENPP1 inhibitor.

## DISCUSSION

In this study, we aimed to identify small molecule agonists of the STING pathway for use as novel anti-tumor immunotherapeutics and host-directed therapy for TB. To this end, we applied a structure-based *in silico* approach to screen an opensource compound library for inhibitors of CDN PDEs from *M.tb* (CdnP) and mammalian cells (ENPP1) that hydrolyze STING agonists such as host 2′,3′-cGAMP and bacterial c-di-AMP. In an earlier study, we tested the FDA-approved PDE-inhibitors cilostazol (PDE3-I), cilomilast (PDE4-I), sildenafil (PDE5-I), and tadalafil (PDE5-I), which demonstrated CdnP inhibition, although at relatively high inhibitor concentrations (23). Subsequently, we designed linear analogs of pApA, the product of c-di-AMP hydrolysis, among which Ap(S)A demonstrated maximal inhibitory activity (*K*_*i*_ = 65 ± 23 µM) against CdnP (23). Treatment with Ap(S)A resulted in a marked elevation of STING-mediated IRF3 activation in uninfected RAW-Blue ISG reporter macrophages transfected with exogenous c-di-AMP or 2′,3′-cGAMP (23). These observations suggested that inhibition of *M.tb* CdnP is experimentally feasible and that CdnP inhibition during *M.tb* infection results in detectable potentiation of cytosolic STING signaling. However, Ap(S)A is a charged, dinucleotide analog that is unlikely to have significant stability or oral bioavailability as a drug *in vivo*.

In this study, we virtually screened the publicly available NCI compound library of about 260,000 molecules for drug-like CdnP inhibitors with low IC_50_ values. We pre-selected 70,000 compounds using the Free ADME-Tox Filtering Tool (Université Paris Cité) to optimize for compounds with favorable pharmacokinetic parameters, low predicted toxicity, and high oral bioavailability. *In silico* screening of compounds against the three-dimensional active site structures of *M.tb* CdnP, as well as the mammalian phosphodiesterase ENPP1, followed by *in vitro* bioactivity studies resulted in the identification of 10 compounds that inhibited *M.tb* CdnP with IC_50_ values of ~43.0 µM (15 µg/mL) or less (five of which showed IC_50_ values of <7.5 µg/mL) and eight compounds that inhibited mammalian ENPP1 with IC_50_ values of ~19.7 µM (15 µg/mL) or less. Most inhibitors were specific for either *M.tb* CdnP or mammalian ENPP1, although a single compound, C-29 (NCI 378695), was active against both enzymes, with IC_50_ values of 14.2 µM (6 µg/mL) for CdnP and 19.4 µM (8.2 µg/mL) for ENPP1. Four of the five most active CdnP inhibitors [IC_50_ < 19.7 µM (<7.5 µg/mL)] and five of the eight most active ENPP1 inhibitors [IC_50_ < 47.0 µM (15 µg/mL)] showed undetectable Vero cell toxicity [IC_50_ for Vero cell growth inhibition of >262 µM (>100 µg/mL)]. Thus, our screen identified 18 inhibitors with potent *in vitro* inhibition against PDEs relevant to STING signaling, nine of which exhibited minimal cellular toxicity.

Recently Karanja et al. reported the identification of inhibitors of c-di-AMP degradation by *M.tb* CdnP using a high throughput screening of 90,000 compounds from the Purdue Chemical Genomics Facility (37). They identified six potent inhibitors, most of which contained oxathieno-pyrimidin- or oxoquinazolin-acetamide moieties, with IC_50_ values ranging from 18 to 60 µM or more (Table 1). By contrast, our compounds, which were identified by computationally predicted binding, feature a greater diversity of scaffold structure and show inhibition values in the low micromolar range. The compounds identified by Karanja et al. specifically inhibited *M.tb* CdnP but did not inhibit ENPP1 or other bacterial CDN PDEs, comparable to the target specificity exhibited by compounds identified in our screen. Together, our studies bolster the potential for further developing novel CDN PDE inhibitors as STING agonist enhancers for TB.

*M.tb* infection triggers the activation of STING signaling via two key mechanisms: by deploying secreted DNA, a substrate for cGAS-mediated production of cGAMP, and by the release of a damage-associated molecule, c-di-AMP, a potent STING agonist (21, 22). We previously reported that *M.tb* shields itself from STING-dependent innate host defenses by deploying CdnP (Rv2837c) to degrade both the bacterial-derived STING agonist c-di-AMP and the host-derived 2′,3′-cGAMP (23). We found that *M.tb* strains that overproduce c-di-AMP or lack CdnP both exhibit significantly reduced virulence in mice, suggesting CdnP as a potential therapeutic target (22, 23). Interestingly, other bacterial PDEs with DHH/DHHA1 or EAL domains, including *Bacillus subtilis* YybT (39), *Staphylococcus aureus* GdpP (40), and *Pseudomonas aeruginosa* RocR (41), exhibit PDE activity toward bacterial CDNs but do not hydrolyze host 2′,3′-cGAMP. Our findings suggest that selective inhibition of *M.tb* CdnP can augment STING signaling via host-produced 2′,3′-cGAMP in addition to the well-characterized bacterial c-di-AMP ligand. Our previous study (23) showed that CdnP functions as a virulence gene that modifies host immune responses in whole animal mouse studies. Indeed, the *M.tb cdnP* knockout mutant showed a significant loss of virulence both by time to death and by organ CFU counts. However, in contrast, intracellular growth of the *M.tb cdnP* transposon mutant (*Mtb-cdnP::Tn*) in bone marrow-derived macrophages (BMDM) showed only a modest (0.3 log) decrease at 5 days compared to *M.tb*-WT, and a potent CdnP inhibitor developed in that study, Ap(S)A (Ki of 65 µM), did not demonstrate *M.tb* killing despite its potent ability to restore levels of STING pathway induction as measured by RAW-Blue ISG reporter macrophages. Similar to our earlier study (23), a limitation of the current work is that while our lead inhibitor, compound E-3, shows *in vivo* potentiation of the STING pathway and elevation of IFN-β levels in macrophages via ENPP1 inhibition (Fig. 6A and B), we have not demonstrated *in vivo* CdnP inhibition or direct *M.tb* killing in the macrophage model. Testing CDN phosphodiesterase inhibitors in *M.tb*-infected macrophages is complicated by the fact that both the host enzyme ENPP1 and bacterial enzyme CdnP are present in the same system; nevertheless, this issue could be addressed in future experiments by using ENPP1 siRNA knockdown or ENPP1-/-BMDMs or alternatively by testing WT versus Mtb-*cdnP*-KO bacteria.

In myeloid cells, cGAS has been shown to serve as an innate immune sensor of DNA products of HIV-1 reverse transcription via a similar mechanism of cGAMP production and STING pathway activation (42). Likewise, STING agonists have been shown to reactivate anti-viral innate immune responses, induce the killing of HIV-1 infected human T cells (43), and reactivate latent SIV in non-human primates with concomitant enhancement of SIV-specific immune responses (44). It is therefore possible that small molecule inhibitors of the CDN PDEs that serve to potentiate STING agonist exposure times may also serve as potent HDTs in the setting of HIV or HIV-TB co-infection.

STING pathway agonism by PDE inhibition likewise shows promise as an approach to broaden the range of tumors that respond to cancer immunotherapy. Since ENPP1 is expressed by both tumor cells and immune cells, small molecule ENPP1 inhibitors could target this enzyme on both cell types, thereby potentiating STING signaling and its antitumor activities in both compartments. This strategy could potentially convert non-inflamed “cold” tumor phenotypes into inflamed “hot” tumor phenotypes that would become more responsive to complementary checkpoint inhibitor therapies. Indeed, in this study, we demonstrated augmented STING activation and IFN I release in response to 2′,3′-cGAMP treatment in murine and human macrophages by one of our lead ENPP1 inhibitor compounds, E-3 (NCI14465) (Fig. 6).

While ENPP1 has been a highly sought-after target in cancer therapeutics, developing drug-like ENPP1 inhibitors has been challenging (45). Both nucleoside-based competitive inhibitors and non-nucleotide-based non-competitive inhibitors of ENPP1 have been generated, but poor bioavailability and potency remain a concern (45, 46). Two key ENPP1 inhibitors under development, SR-8314 and MV-626, have exhibited antitumor efficacies in animal models (47, 48). However, given the concern of hyperinflammation due to heightened STING activation following ENPP1 inhibition, the optimal dose and duration of treatment for ENPP1 inhibitors have yet to be established, and the search for potent and safe ENPP1 inhibitors continues. More recently, Carozza et al. (49) carried out elegant structure-activity relationship studies and identified numerous potent ENPP1 inhibitors with *K*_*i*_ in the range of <2–33 nM . Notably, their scaffolds differ from the compounds we describe here, further demonstrating the broad chemical landscape of ENPP1 inhibitors. Hence, for the ENPP1 inhibitors identified in our study to be valuable for TB therapy, our focus of future preclinical *in vitro* and *in vivo* studies will be to establish optimal drug concentrations, duration of treatment, and efficacy in combination with standard anti-mycobacterial therapies in preclinical *in vitro* and *in vivo* studies in order to support transient increases of the type I IFN response and minimize long-term hyperinflammation.

In summary, this study reveals novel inhibitors of two phosphodiesterase enzymes known to inactivate CDN STING agonists: *M.tb* CdnP and mammalian ENPP1. Eighteen small molecule inhibitors with good *in vitro* IC_50_ values were identified, and nine of these showed low-level toxicity in Vero cells. We validated our lead ENPP1 inhibitor (E-3, NCI 14465) in mammalian macrophages and demonstrated proof of principle potentiation of STING signaling. These agents show potential promise toward the development of novel host-directed therapies for TB and other paradigms that may benefit from enhanced STING signaling, including tumor immunotherapy.

## MATERIALS AND METHODS

### *In silico* screening against ENPP1 and CdnP

*In silico* screening of compound libraries for potential inhibitory activity against ENPP1 and CdnP was conducted using structure-based virtual screening. Briefly, three-dimensional structures of 260,071 compounds available from the NCI Open database library (https://dtp.cancer.gov) were downloaded as .sdf files and subsequently converted to .smi format and filtered using various physiochemical parameters, including the Lipinski rule of five, ADME parameters, and predicted toxicity, as previously described (50). Parallel grid computing using AutoDock 4.2 software was performed using the Maryland Advanced Research Computing server (https://www.marcc.jhu.edu). The customized library was uploaded onto the server along with the minimized 3D PDB structures of CdnP (38) and ENPP1 (17). A genetic algorithm with default parameters was employed for the docking calculations. Compounds were sorted by decreasing score of binding affinity, and the top 80 molecules against each of CdnP and ENPP1 that were available on the NCI website were procured. Select freely available compounds for further screening were obtained from the Drug Synthesis and Chemistry Branch, Developmental Therapeutics Program, National Cancer Institute, National Institutes of Health, Bethesda, MD, USA.

### Expression of *M.tb* CdnP and purification

The CdnP-encoding gene of *M. tuberculosis*, *MT2903* (*Rv2837c*), was cloned into the *E. coli* expression vector pET32a and subsequently used to transform *E. coli* BL21 DE3 strain, from which the expressed protein purified as described earlier (23). Human ENPP1 protein was purchased from Origene.

### *In vitro* enzyme assays

CdnP hydrolyzes c-di-AMP into AMP and pApA, while ENPP1 hydrolyzes 2′,3′-cGAMP into AMP and GMP. All the substrate and product species resolved well in an HPLC/UPLC C18 column. In our HPLC system, c-di-AMP elutes at 17.87 minutes, pApA at 16.04 minutes, and AMP at 7.83 minutes, thus showing a great resolution for the substrates as well as products. Enzyme kinetic experiments were conducted with CdnP and ENPP1 enzyme concentrations of 5 µM and increasing concentrations of c-di-AMP or 2′,3′-cGAMP, and product peaks were quantified with known standards. Since HPLC does not serve well for high throughput analysis, a luminescence-based AMP-Glo assay (Promega) was used to measure AMP produced in the reactions using the Promega luminometer in a 96-well format assay. Briefly, the assay quenches ongoing enzymatic reactions and depletes any pre-existing ATP. Subsequently, the AMP product is converted to ATP, thus fueling the production of luminescence by a luciferase reaction in proportion to the concentration of AMP. The resulting relative light unit measurements are thus directly used to extract IC_50_ parameters. Using this luminescence-based assay, enzyme kinetic experiments were conducted under 60:1 substrate-excess conditions (c-di-AMP or 2′,3′-cGAMP substrates at 300 µM and CdnP or ENPP1 enzymes at 5 µM). Inhibitor concentrations ranging from ~2.5 to ~300 µM (0.78–100 μg/mL) were used to determine IC_50_ values.

### Determination of IC_50_ against ENPP1 and CdnP

The compounds procured from NCI-DTP were dissolved in dimethyl sulfoxide (DMSO) to a stock concentration of 5 mg/mL. Compounds at 100 µg/mL were incubated with CdnP or ENPP1 at 5 µM for 30 minutes, at which point the respective CDN substrate was added. This reaction was allowed to incubate for 2 h before the addition of AMP-Glo kinase assay kit (Promega) reagents. The plates were read in the Promega luminometer 30 minutes after AMP-Glo reagent addition. Purified AMP was used as a standard. Compounds exhibiting more than 30% inhibition at 100 µg/mL were further titrated using twofold dilutions ranging from 100 to 0.78 μg/mL to determine the IC_50_ values. The percentage inhibition was calculated accordingly and plotted with GraphPad Prism (v9).

### Vero cell cytotoxicity

Cytotoxicity of the compounds against Vero cells (ATTC CCL-81) was measured by a resazurin dye reduction assay. In the presence of functional mitochondrial respiration, the blue dye resazurin (7-hydroxy-3H-phenoxazin-3-one-10-oxide) is reduced to the pink, highly fluorescent resorufin. This reduction can be quantified via fluorescence spectroscopy with peak absorption of resazurin at 600 nm and resorufin at 570 nm, with conversion to the latter in proportion to cell viability. Vero cells were cultured in Dulbecco’s Modified Eagle Medium (DMEM) with 10% fetal bovine serum (FBS) and antibiotic and antimycotic mix in a 96-well plate for 24 h. After 24 h, cells were washed with sterile phophate-buffered saline (PBS) and incubated in DMEM containing varying concentrations of the inhibitors (0.36–100 μg/mL), with rifampicin and DMSO as negative controls. After 48 h of incubation, resazurin was added to each well and incubated overnight. The absorbances at 570 and 600 nm were measured using the FLUOstar Optima fluorescence microplate reader (BMG Labtech Inc.).

### Cytochrome P450 studies

The inhibition of CYP2C19 activity was determined using the P450-Glo assay (Promega) according to the manufacturer’s instructions. Briefly, purified recombinant human CYP2C19 isozyme was purchased from Promega. The enzyme was incubated with 100 µg/mL of each compound for 30 minutes, followed by the addition of the specific luminescent substrate and the NADPH regenerating system (containing NADP+, glucose-6-phosphate, MgCl2, and glucose-6-phosphate dehydrogenase) provided with the kit. The reaction mixture was incubated at room temperature for 30 minutes followed by the addition of the luciferin detection reagent. The luminescence was measured using a luminometer after incubation with the reagent for 20 minutes.

### Evaluation of 2′,3′-cGAMP-mediated type I IFN responses in murine and human macrophages following treatment with ENPP1 inhibitor

RAW-Blue macrophage reporter cells were previously derived from the murine RAW264.7 macrophage cell line via stable transfected with IRF-inducible alkaline phosphatase (SEAP) reporter construct (InvivoGen). The cells were maintained in DMEM supplemented with 10% (vol/vol) heat-inactivated fetal bovine serum, penicillin (100 U/mL), streptomycin (100 µg/mL), normocin (100 µg/mL), and zeocin (200 µg/mL). The cells were routinely tested for mycoplasma contamination while the cells were in culture and were grown for not more than 10 passage cycles. Human primary monocyte-derived macrophages were generated using peripheral blood-derived mononuclear cells (PBMCs) isolated from healthy male donors (leukopacks) aged between 18 and 30 years. Briefly, to separate blood constituents and isolate the buffy coat, density gradient centrifugation (400 × *g* at 18°C for 30 minutes) was performed on blood diluted in RPMI-1640 over Ficoll-Paque Plus reagent (Cat. 17-1440-02, GE Healthcare, Piscataway, NJ, USA). The cells were washed several times using 1× PBS and counted for viability. Following viability testing, CD14^+^ human monocytes were enriched from whole PBMCs using magnetic labeling (Monocyte Isolation Kit II, Cat. 130-091-153, Miltenyi Biotec, San Diego, CA, USA). The purity of isolated CD14^+^ cells was confirmed using a fraction of cells stained with a fluorochrome-conjugated antibody against a monocyte marker (as recommended by the manufacturer) and subsequently analyzed using a BD-LSR2 flow cytometer (data not shown). Human monocytes were seeded at 2.0–3.0 × 10^5^ cells/mL in RPMI 1640 medium supplemented with 10% FBS and 1% streptomycin/penicillin at 37°C with 5% CO_2._ Monolayers of CD14^+^ monocytes were differentiated into M1 [GM-CSF (20 ng/mL, PeproTech, Rocky Hill, NJ, USA) and IFN-γ (20 ng/mL, PeproTech, Rocky Hill, NJ, USA )] macrophages for 7 days.

### *In vitro* drug treatment and transfection assays

Studies of ENNP1 inhibitors on the STING/IRF3/IFN I pathway were achieved by the transfection of 2′,3′-cGAMP-sodium salt (Cayman, item no. 19887) into RAW-Blue reporter macrophages and M1 polarized PBMC-derived primary human macrophages. Briefly, 0.2 × 10^6^ macrophages were seeded for attachment for 4 h following an overnight incubation in the presence of varying concentrations of ENPP1 inhibitors in 96-well tissue culture plates. Following overnight incubation, cells were repeatedly washed using Dulbecco’s PBS to remove any traces of drug prior to 2′,3′-cGAMP transfection. For *in vitro* transfection of 2′,3′-cGAMP, cells were incubated for 2 h in the presence of reduced serum (2% FBS) containing DMEM followed by X-tremeGENE9-mediated transfection for 24 h. Culture supernatants were collected from RAW-Blue cells for the estimation of IRF induction by SEAP colorimetric assay QUANTI-Blue reagent (InvivoGen) and from primary PBMC-derived hMDMs for IFN-β ELISA.

### ELISA

Culture supernatants from 2′,3′-cGAMP-transfected primary hMDMs were collected and filter-sterilized to remove debris. Sandwich ELISA was performed using the VeriKine-HS Human IFN-β ELISA kit to probe interferon-β concentrations.

## SUPPLEMENTAL MATERIAL

Supplemental figures spectrum.02012-23-s0001.pdf Figures S1 to S3.

ASM does not own the copyrights to Supplemental Material that may be linked to, or accessed through, an article. The authors have granted ASM a non-exclusive, world-wide license to publish the Supplemental Material files. Please contact the corresponding author directly for reuse.
