# Supplementary material for: Structure-based virtual screening and in vitro validation of inhibitors of cyclic dinucleotide phosphodiesterases ENPP1 and CdnP
Source: Microbiol Spectr. 2023 Dec 14;12(1):e02012-23. doi: 10.1128/spectrum.02012-23 (PMC10783014; doi:10.1128/spectrum.02012-23)
Supplement: Supplemental figures — Figures S1 to S3. [file spectrum.02012-23-s0001.pdf]

Supplemental Figure 1

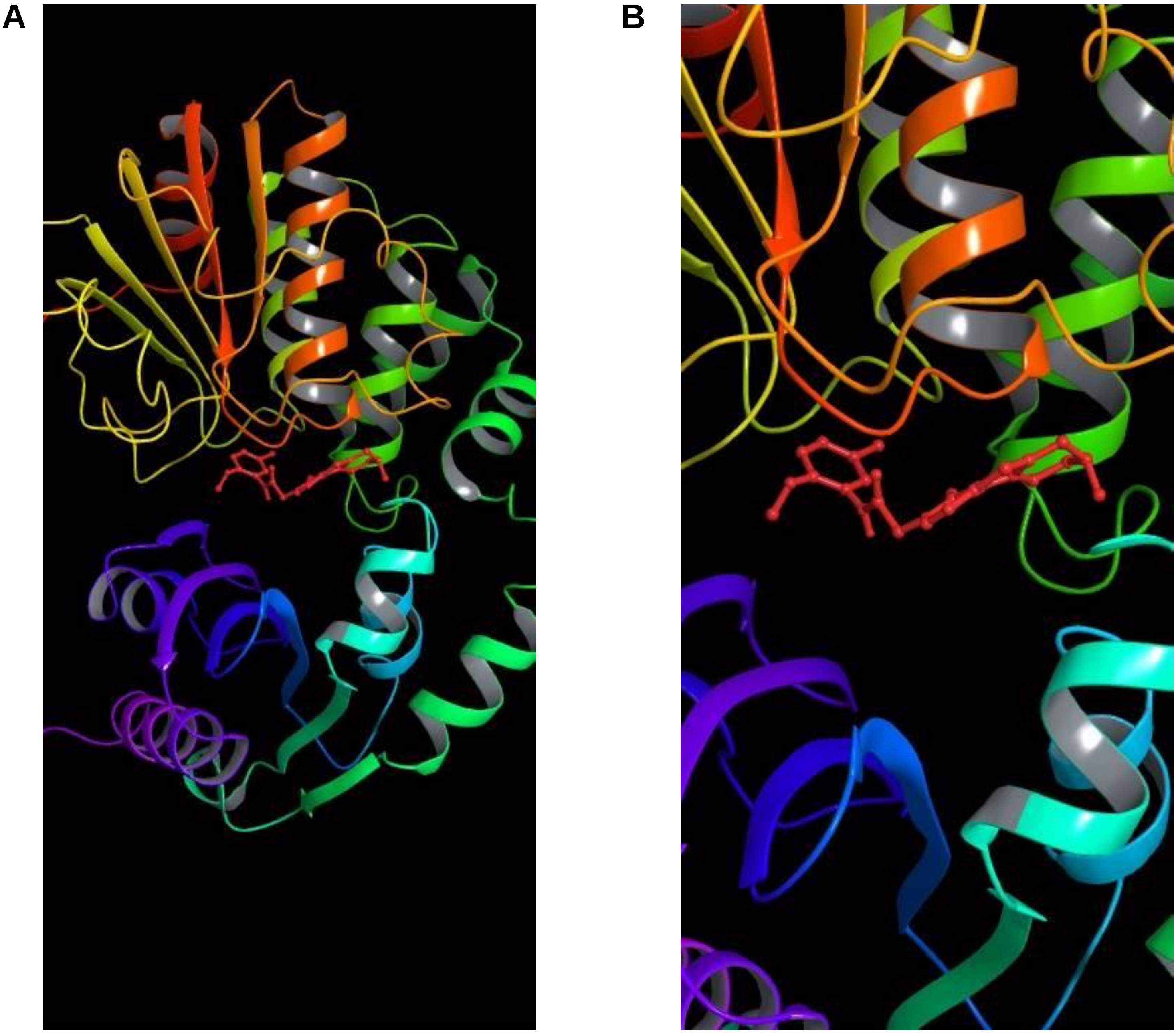

Supplemental Figure 1: **Virtual reproduction of the CdnP enzyme ribbon structures with the Karanja et al. 2021 inhibitor C85 docked at the active site. A.** Low and **B**. high magnification of Karanja et al. 2021 inhibitor C85 (red) docking to the active site of CdnP. C85 had a docking score of -8.07 kcal/mol and a CdnP IC_{50} value of 22.0 μM.

Supplemental Figure 2

Additional CdnP inhibitors

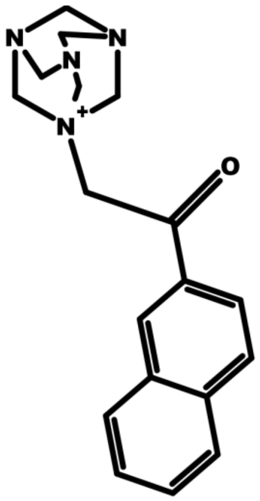

C-5. NCI 36400.
CdnP IC_{50} 28.8 μM
(11.2 μg/ml)

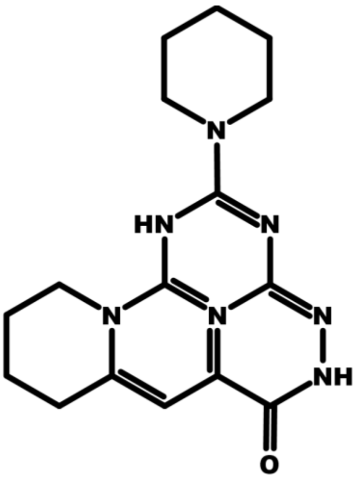

C-15. NCI 99864.
CdnP IC_{50} 21.5 μM
(8.2 μg/ml)

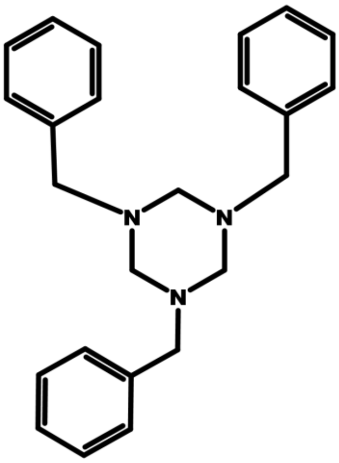

C-67. NCI 169717.
CdnP IC_{50} 41.9 μM
(15 μg/ml)

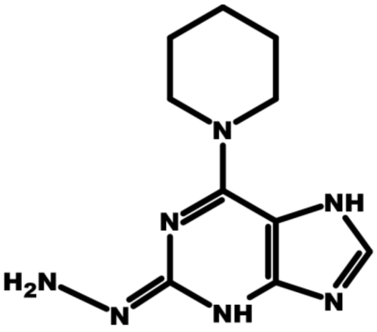

C-76. NCI 401266.
CdnP IC_{50} 42.9 μM
(10 μg/ml)

Additional ENPP1 inhibitors

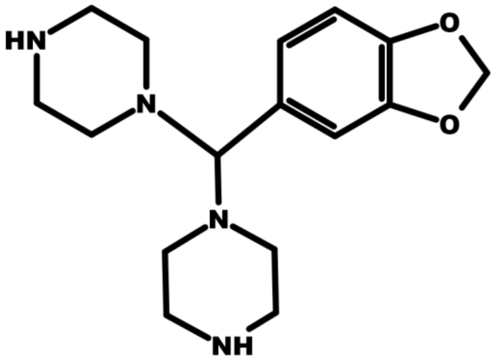

E-12. NCI 87015.
ENPP1 IC_{50} 41.1 μM
(12.5 μg/ml)

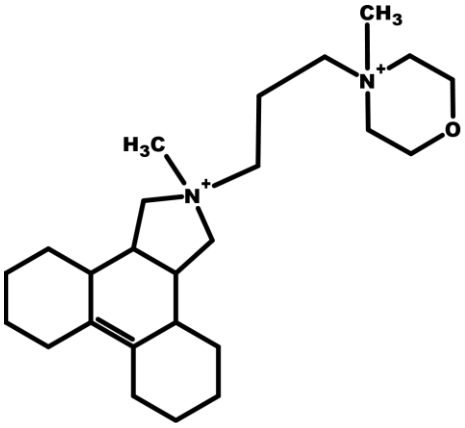

E-17. NCI 107121.
ENPP1 IC_{50} 15.6 μM
(10 μg/ml)

Supplemental Figure 2: Structures, NCI numbers, and IC_{50} values for additional CdnP and ENPP1 inhibitors

Supplemental Figure 3

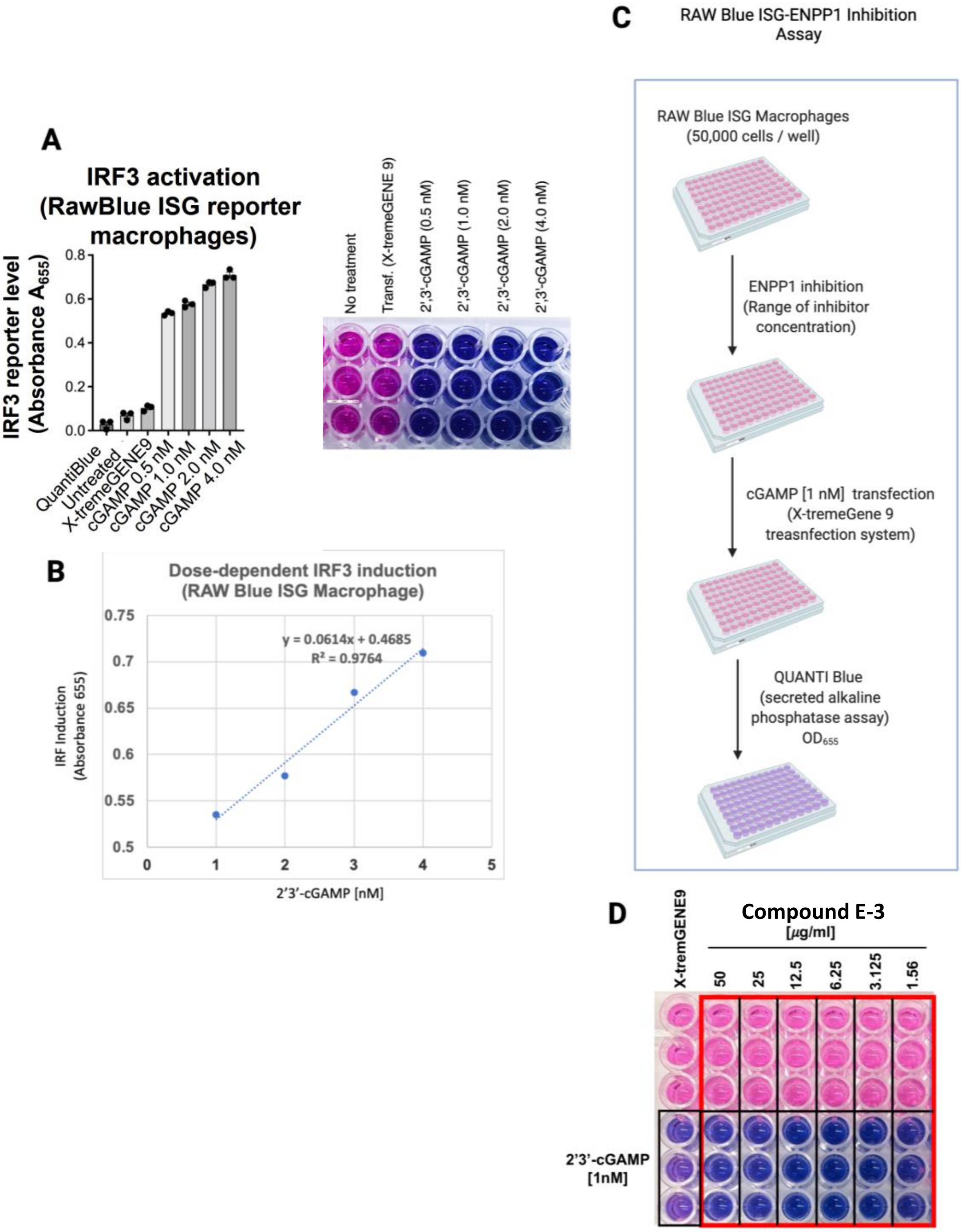

Supplemental Figure 3. IRF3 pathway induction measured by IRF-SEAP QUANTI Blue reporter assay. A. Bar graph showing increased IRF3 induction in RAW Blue ISG macrophages in response to 2',3'-cGAMP transfection. Representative image of QUANTI Blue assay plate and sample wells corresponding to the bar graph. **B.** Linear relationship between A_{655} and increasing concentrations of 2',3'-cGAMP. **C.** Schematic diagram showing RAW Blue ISG-ENPP1 inhibition assay. Briefly, 2',3'-cGAMP transfection was achieved using X-tremGENE9 transfection reagent to stimulate cGAS/STING/IRF3 activation. **D.** Representative images of induction of IRF3 by ENPP1 inhibition with lead compound E-3 (NCI 14465). ENPP1 inhibition-mediated increased IRF3 induction was determined by pre-treating RAW Blue ISG macrophages with compound E-3 before 2',3'-cGAMP transfection. Related to **Fig. 6**.
